# Quantitative, Non-Disruptive Monitoring of Transcription in Single Cells with a Broad-Host Range GFP-*luxCDABE* Dual Reporter System

**DOI:** 10.1371/journal.pone.0052000

**Published:** 2012-12-28

**Authors:** Ilaria Maria Benedetti, Victor de Lorenzo, Rafael Silva-Rocha

**Affiliations:** Systems Biology Program, Centro Nacional de Biotecnología CSIC, Cantoblanco-Madrid, Spain; Center for Genomic Regulation, Spain

## Abstract

A dual promoter probe system based on a tandem bi-cistronic GFP-*luxCDABE* reporter cassette is described and implemented. This system is assembled in two synthetic, modular, broad-host range plasmids based on pBBR1 and RK2 origins of replication, allowing its utilization in an extensive number of gram-negative bacteria. We analyze the performance of this dual cassette in two hosts, *Escherichia coli* and *Pseudomonas putida*, by examining the induction properties of the lacI^q^-*Ptrc* expression system in the first host and the *Pb* promoter of the benzoate degradation pathway in the second host. By quantifying the bioluminescence signal produced through the expression of the *lux* genes, we explore the dynamic range of induction for the two systems (*Ptrc*-based and *Pb*-based) in response to the two inducers. In addition, by quantifying the fluorescence signals produced by GFP expression, we were able to monitor the single-cell expression profile and to explore stochasticity of the same two promoters by flow cytometry. The results provided here demonstrate the power of the dual GFP-*luxCDABE* cassette as a new, single-step tool to assess promoter properties at both the population and single-cell levels in gram-negative bacteria.

## Introduction

Living organisms are equipped with complex machinery dedicated to interaction with the surrounding environment. In fact, significant portions of prokaryotic genomes encode genes and elements associated with the processes of signal transduction and gene regulation, and the number of these elements is relatively large for generalist organisms [1]. Among the different constituents of the intricate regulatory networks in living cells, the promoters have a central role in the process of gene expression [2], [3]. The promoter sequence marks the physical region of the genome where both the expression apparatus (i.e., the RNA polymerase) and the regulatory elements (represented by transcriptional factors) are recruited in order to control the process of RNA production [2], [3]. In this sense, most of the signal integration process occurs at this specific spot through the interplay of a few to many elements (usually proteins), its final result being the control of the expression of the target gene [1], [3], [4]. However, the intrinsic nature of this process makes it prone to stochastic fluctuations that could affect its final outcome (i.e., the number of output molecules produced under a particular condition). The level of stochasticity in gene regulation is mainly related to the reaction rate between the components of the transcription initiation machinery when they are in short supply [5]–[7]. The phenotype observed at the macroscopic scale using classical approaches (such as promoter analysis using enzymatic reporters e.g. β-galactosidase activity measurement) only represents the average behavior of the whole. But such population-wide measurements of promoter activity say nothing on the performance of given promoters in individual cells, which can vary dramatically depending on the specific regulatory network [8]–[11]. Yet, information on individual transcriptional activity and how it relates to the properties of the population as a whole is crucial for understanding the basic mechanisms underlying the gene expression process [8], [9], [11], [12].

Currently, most available methodologies for assessing the effects of stochastic processes in single cells are based on the use of a fluorescent reporter gene (typically GFP or similar) fused to the target promoter element [13]–[15]. Thus, the observation of changes in the fluorescence signals during experiments, mainly through time-lapse microscopy or flow cytometry, allows the investigation of stochastic processes that are intrinsic to the operation of regulatory networks in response to perturbation [9], [16]. However, fluorescent reporters may provide limited information under some specific conditions when the population behavior is the object of interest [17]. Thus, for these particular conditions, a more robust methodology would be required to assess the dynamics of gene regulation in response to an external stimulus. A powerful technology that allows the execution of high-throughput experiments is represented by the bioluminescence-emitting *lux* reporters [18]. The *lux* systems works by converting cellular reducing power into a light signal that can be easily quantified [19]. These systems provide much higher resolution than traditional systems, as a lower background signal is typically autonomously generated by the host, and thus allow a more accurate quantification of changes in promoter activity in response to a given stimulus [18], [19]. However, most bioluminescence-emitting reporter systems are problematic because of their inherent dependence on the energetic status of the cell for their proper activity. This drawback of *lux*-related reporters is nonexistent with fluorescence-based systems such as GFP, the readout of which is virtually independent on the metabolic status of the cell. Additionally, *lux*-based systems are not suitable for examining stochastic processes at the single-cell level, as the signal produced is diffusive [19]. In this context, an ideal promoter parameterization system should combine the strengths of these two reporters to allow the execution of single-cell experiments with the added capability of performing high-throughput experiments at high resolution within a simplified promoter cloning platform. Since such an ideal reporter is not yet available, a useful alternative is to artificially combine the two systems (GFP and *lux*) into a synthetic single transcriptional unit. A similar approach has been successfully applied for monitoring promoter activity in gram-positive bacteria [20]. However, the system was assembled with naturally occurring DNA segments which limited the number of restriction enzymes suitable for promoter cloning [20]. Furthermore, the value of a dual GFP-*lux* cassette in gram-negative bacteria for transcriptional studies is uncertain, as it has been limited thus far to tagging cells for ecological experiments [21], [22].

In this report, we present a new dual reporter cassette based on the *gfp* and *luxCDABE* systems that is suitable and optimized for promoter probing and parameterization in gram-negative bacteria. The system is implemented in two modular, synthetic broad-host range vectors based on the pBBR1 and RK2 origins of replication. As explained below, the DNA segment encoding the upstream 5′ boundary of the dual reporter genes was engineered with an expanded set of unique restriction sites that facilitate the cloning and analysis of a wide variety of promoters. These vectors enable examination of stochastic transcription phenomena in an extensive range of bacterial hosts [23], thereby helping to fill the existing gap in promoter analysis tools for gram-negative bacteria other than *E. coli*.

## Results and Discussion

### Rationale and Design of a Dual GFP-*luxCDABE* Reporter System

**Figure 1:**
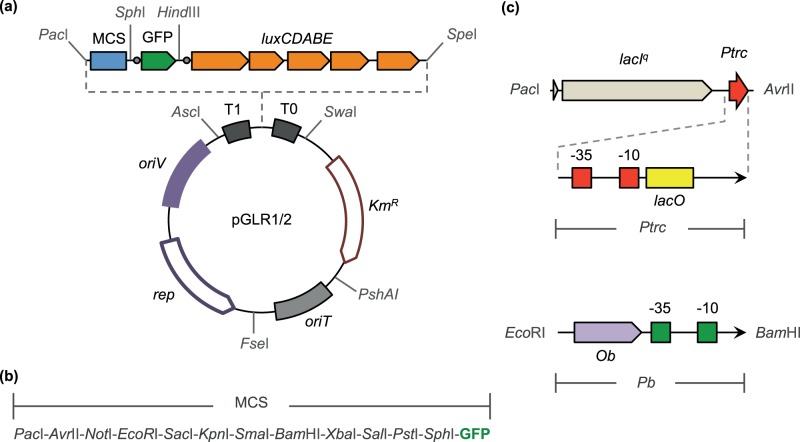
Structural organization of pGLR1/2 vectors and assayed expression systems. (**a**) The vectors each harbor a kanamycin resistance (Km^R^) marker, an *oriT* for plasmid transfer through conjugation and a broad-host range origin of replication that consists of a vegetative origin (*oriV*) and a replication protein (*rep*). Vector pGLR1 is based on a minimal pBBR1 origin [41], while pGLR2 is based on ori RK2 [42]. The GFP*-luxCDABE* reporter cassette is cloned between two strong terminators (T0 and T1) and is downstream of a multiple cloning site (MCS). The optimal ribosome-binding site of the TIR element (represented as a grey circle [24]) is placed upstream of the *gfp* gene and the first gene of the *lux* operon (*luxC*). (**b**) List of enzymes found at the MCS. (**c**) Architecture of the *lacI^q^-Ptrc* expression system (top) and the *Pb* promoter (bottom). Relevant features such as operators (*lacO* and *Ob*) and promoter (−10/−35) regions are represented.

To create a new dual reporter cassette, we considered three fundamental elements. First, we used a previously described GFP variant with an optimized ribosome binding site (GFP*tir* [24]). Second, we used a complete *lux* operon from *Photorhabdus luminescens* (*luxCDABE*) that encodes all the enzymes necessary to convert cellular reducing power to a luminescent signal [18]. Finally, we considered a delivery system that is compatible with a large number of gram-negative hosts. In this sense, we implemented the reporter system in a new set of minimal, synthetic, broad-host range vectors based on the pSEVA format (http://seva.cnb.csic.es, [25]) generating the pGLR1 and pGLR2 vectors (Fig. 1a). SEVA vectors are arranged with a fixed format that includes three synthetic functional modules (the *ori* of replication, the antibiotic marker and the *cargo* segment) flanked by unique, unusual restriction sites that facilitate interchangeability between different components. Functional modules of SEVA plasmids are edited for erasing any of the restriction sites of the pUC18 polylinker as thus ease cloning operations [25]. The dual GFP*-lux* cassette system was assembled into two vectors, each harboring a kanamycin resistance marker and either a pBBR1- (in the case of pGLR1) or RK2- (for pGLR2) based origin of replication, both of which are known to replicate in a wide number of hosts [23]. These vectors each present a modular architecture that facilitates the interchange of functional modules (such as the antibiotic resistance marker) to produce new variants if required. As shown in the Fig. 1a, the *lux* genes are cloned between *Hind*III and *Spe*I restriction sites of the plasmid vectors, as in the *lux*-based promoter probe plasmid pSEVA226 [25]. But, in contrast, the GFP*tir* gene, encoding a stable variant of GFP, was placed upstream of the *lux* operon between *Sph*I and *Hind*III restriction sites. It is important to note that since this GFP is stable, the protein half-life will generally equal the doubling time of the cell when the accumulated reporter is diluted between the two progeny cells. It is also worth noting that both reporter systems use the same optimized ribosome-binding site (the TIR element [24]) preceding the start codon. In the case of *lux*, the TIR element is located upstream of the first gene of the operon (*luxC*). The resulting dual cassette is located downstream of an expanded multiple cloning site (MCS) composed of 12 unique restriction sites that are used for promoter cloning (Fig. 1b). An identical *gfp-lux* dual construct vector bearing an unstable GFP protein (*gfplva*, with an half-life of ∼15 minutes) was engineered as well for transient expression studies [26] (Table 1).

**Table 1:** Bacterial strains and plasmids used in this work.

Strains/Plasmids	Genotypes or description	Reference
*E. coli*		
CC118	Δ*(ara-leu) araD* Δ*lacX74 galE galK phoA thi1 rspE rpoB argE (Am) recA1n*	[47]
HB101	*rpsL* (Sm^R^), *recA, thi, pro, leu, hsdR*- *hsdr*+ (*E. coli* K12/*E. coli* B hybrid)	[48]
MG1655	Prototrophic, *recA^+^,* reference K12 strain	[27]
*P. putida*		
KT2440	*rsdR*; *P. putida* mt-2-derivative cured of the pWW0 plasmid	[49]
MEG3-*Pb*	Sm^R^, Rif^R^; *P. putida* MEG3 derivative with chromosomal insertion of a *Pb::*GFP-*lacZ* fusion	[30]
*Plasmids*		
pRK600	Cm^R^, *oriColE1, mobRK2, traRK2*; helper for mobilization of *oriT RK2*+*-*containing plasmids	[50]
pSEVA221	Km^R^, *oriRK2, oriT*; standard broad-host-range plasmid for Gram-negative bacteria	[25]
pSEVA224	Km^R^, *oriRK2, oriT*; pSEVA221-derivative with *lacI^q^/Ptrc* expression system	[25]
pSEVA236	Km^R^, *oripBBR1, oriT*; standard broad-host-range with *luxCDABE* reporter system	[25]
pGreenTIR	Ap^R^, *oriColE1*; promoterless cloning vector with *gfp tir* gene	[24]
pGLR1	Km^R^, *ori pBBR1, oriT*; pSEVA236-derivative with dual GFP*-luxCDABE* reporter system	This work
pGFLR1	Km^R^, same than pGLR1 but encoding the short-lived *gfplva* variant [26]	This work
pGLR2	Km^R^, *oriRK2, oriT*; pSEVA221-derivative with dual GFP*-luxCDABE* reporter system	This work
pGLR1-*Ptrc*	Km^R^, *ori pBBR1, oriT*; pGLR1-derivative with *lacI^q^/Ptrc* expression system cloned as a *Pac*I/*Avr*II fragment	This work
pGLR2-*Pb*	Km^R^, *oriRK2, oriT*; pGLR2-derivative with *Pb* promoter cloned as a *Eco*RI/*Bam*HI fragment	This work
pLUX-*Ptrc*	Km^R^, *oriRK2, oriT*; pSEVA224-derivative with the *luxCDABE* operon cloned as a *Hind*III/*Spe*I fragment	This work
pGFP-*Ptrc*	Km^R^, *oriRK2, oriT*; pSEVA224-derivative with *gfptir* cloned as a *Hind*III/*Spe*I fragment	This work
pGLR1-*PlexA*	Km^R^, *ori pBBR1, oriT*; pGLR1-derivative with *PlexA* promoter cloned as a *Eco*RI/*Bam*HI fragment	This work
pGFLR1-*PlexA*	Km^R^, same than pGLR1-*PlexA* but encoding the short-lived *gfplva* variant [26]	This work

**Figure 2:**
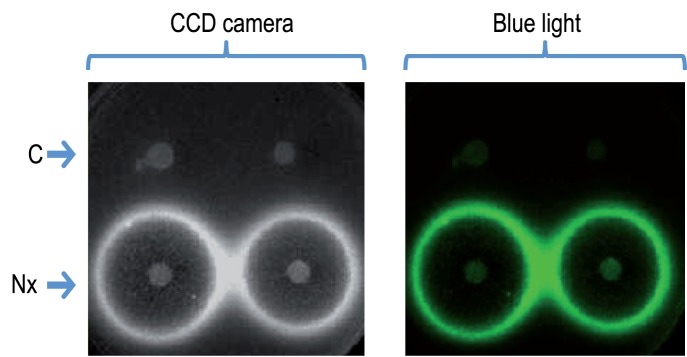
Co-occurrence of *lux* and *gfp* activity in a plate assay. Strain *E. coli* MG1655 (pGLR1-*PlexA)* was added to top agar and overlaid onto an LB plate with kanamycin for plasmid retention. Disks containing 25 ng of nalidixic acid (or controls with water) were then deposited onto the surface, and the overnight-grown plates were processed to reveal either bioluminescence with a CCD camera or fluorescence with a blue light as explained in Materials and Methods. Note the coincidence of both images with different reporters.

To initially determine the value of the system for reporting promoter activity in response to a known signal, we inserted a PCR fragment spanning the SOS promoter *PlexA* of *E. coli,* which responds to exogenous damage to DNA [27], into pGLR1. The resulting construct pGLR1-*PlexA* was then transformed in *E. coli* strain MG1655 and the cells overlaid onto a plate with nalidixic-acid containing disks. After growth, the plate was then either exposed to blue light (for revealing GFP) or examined in the dark with a CCD camera for capturing light emission. The images shown in Fig. 2 display a boundary concentration of the quinolone antibiotic at which cells express *PlexA* at a high level, which is bound by either growth inhibition or by virtually no activity of the same promoter. Furthermore, the two images of fluorescence and luminescence overlapped perfectly, thereby demonstrating their coincidence in time and space and the lack of internal promoters able to create an artifactual background. When a similar plate test was conducted with strain *E. coli* MG1655 (pGFLR1-*PlexA*), which expresses a short-lived GFP protein, (Table 1) similar images were obtained, although the intensity of the fluorescence signal was, expectedly, lower (not shown).

### Co-occurrence of GFP and *luxCDABE* in the Same Expression Unit

One key requirement of any robust dual reporter is the good correspondence between the readouts stemming from each of the cognate gene products. To examine this issue in the GFP-*luxCDABE* cassette described above, the system was assayed in two hosts, *E. coli* and *P. putida*, using the *lacI^q^-Ptrc* expression device [28] and the *Pb* promoter of the benzoate degradation pathway from *P. putida* [29], [30], respectively (Fig. 1c). It is worth noting that, to validate the two vectors presented here, the *lacI^q^-Ptrc* expression system was assayed in the pGLR2 vector (RK2-based) while *Pb* was analyzed in pGLR1 (pBBR1-based).

**Figure 3:**
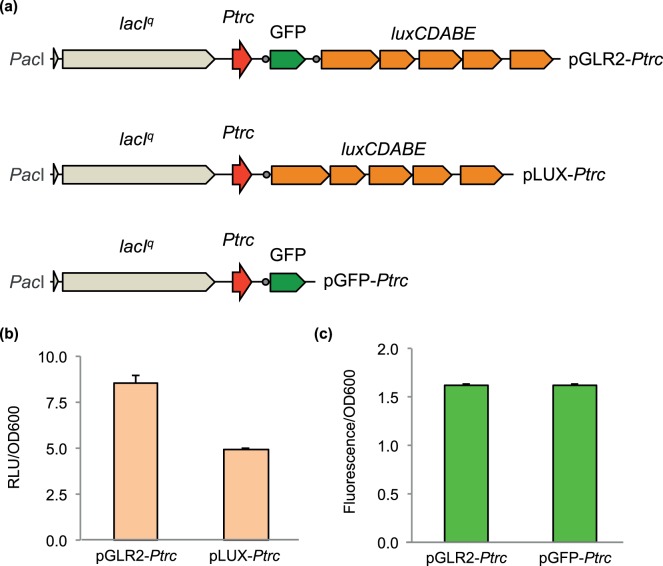
Validation of the dual GFP*-luxCDABE* reporter system. (**a**) Structure of the reporter systems used. In the three cases, the expression of the different reporters is triggered by a *lacI^q^-Ptrc* expression system [28]. Plasmid pGLR2-*Ptrc* has the dual reporter system, while in pLUX-*Ptrc*, only the *luxCDABE* operon is present. Additionally, in the pGFP-*Ptrc* plasmid, GFP alone is placed under the control of the *lacI^q^-Ptrc* system. (**b**) and (**c**) Comparison of reporter performances in response to IPTG. Overnight-grown cells were diluted 1∶20 in fresh M9 minimal media with 1 mM of IPTG. After 4 h of induction, promoter activities were calculated by normalizing the reporter signal (RLU or fluorescence) to the OD_600_. In this sense, promoter activity represents RLU/OD_600_ in (**b**) and fluorescence/OD_600_ in (**c**). Vertical bars represent the standard deviation calculated from four technical replicates.

To characterize the performance of the *GBF-lux* cassette, we first investigated the effect of concatenating the *gfp* and the *lux* reporters into a single transcriptional unit. For this assessment, we compared the promoter output of the *Ptrc*-based system in *E. coli* with each reporter alone compared to the dual cassette. A schematic representation of the transcriptional fusions created is shown in Fig. 3a. To assay promoter activity, *E. coli* strain CC118 was transformed with pGLR2-*Ptrc* (*Ptrc*::GFP-*luxCDABE* fusion), pLUX-*Ptrc* (*Ptrc*::*luxCDABE*) or pGFP-*Ptrc* (*Ptrc*::GFP) and assayed in M9 minimal medium with glucose and casamino acids in the presence of 1 mM of IPTG. As shown in Fig. 3b, the insertion of the *gfp* gene between the *Ptrc* promoter and the *lux* operon had a moderate positive effect on the bioluminescence signal, as the final promoter activity was ∼1.75 times higher in cells harboring the pGLR2-*Ptrc* plasmid than in cells harboring pLUX-*Ptrc*. Furthermore, a comparison of promoter activity using the fluorescent reporter shows no difference between plasmids pGLR2-*Ptrc* and pGFP-*Ptrc* (Fig. 3c), suggesting that the presence of the *gfp* gene upstream of the *lux* operon generated no interference with the transcription/translation of the later. These results demonstrate that the dual GFP-*luxCDABE* cassette implemented here works as a fully functional polycistronic unit that is suitable for monitoring promoter activity using both fluorescence and bioluminescence emissions.

### Assessing Inducer-dependent Kinetics Using the Bioluminescent Reporter

**Figure 4:**
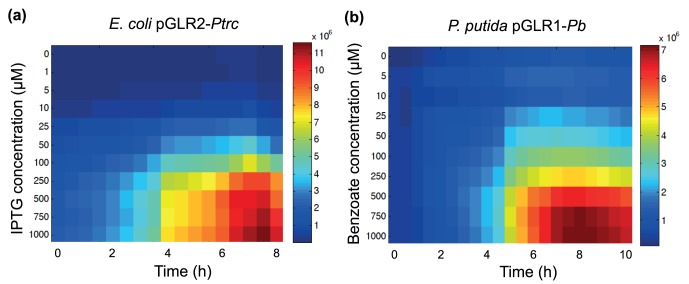
Expression landscape using bioluminescence signal in *E. coli* and *P. putida* hosts. Overnight-grown strains were diluted 1:20 in fresh media containing different concentrations of IPTG or benzoate, as indicated. At 30 min intervals, bioluminescence and OD_600_ signals were recorded. Figures represent the level of promoter activities for each strain relative to time. *E. coli* CC118 harboring pGLR1-*Ptrc* (**a**) was induced with IPTG, while *P. putida* KT2440 with pGLR2-*Pb* was induced with benzoate (**b**). Color bars at left represent the scale for promoter activity.

As mentioned before, among the principal advantages of *lux* reporters is their ability to perform high-throughput experiments to assay numerous conditions. This benefit is apparent, for example, in experimental designs used to determine the induction kinetics of a given expression system in response to different concentrations of inducers. This type of experiments is crucial for determining the exact transfer-function for a particular regulatory node [31]. To evaluate the performance of the GFP-*luxCDABE* cassette in measuring promoter induction kinetics, we analyzed the expression of the *Ptrc*- and *Pb*-based constructs in response to their cognate inducers in *E. coli* and *P. putida*, respectively. These two systems display some of the most common mechanisms of gene regulation in bacteria. In the first system, the LacI protein represses *Ptrc* activity by blocking the binding of the RNA polymerase (RNAP) to this promoter. Transcription thus occurs when the inducer (lactose, IPTG, etc.) binds to LacI and removes it from *Ptrc*, allowing initiation [28]. In the case of *Pb*, a transcriptional activator, BenR, triggers the promoter activity when bound to the inducer benzoate. This regulator belongs to AraC-family and works by recruiting RNAP to the target promoter [29]. By separately fusing each of these two promoters to the GFP-*luxCDABE* reporter, we assayed both transcriptional repression and activation in the two model organisms. For these tests, overnight-grown cells were diluted in fresh media containing increasing concentrations of the specific inducer (IPTG or benzoate) and assayed in a multilabel plate reader, as described in Material and Methods. At time intervals of 30 min, the optical density at 600 nm and the luminescence were recorded. Fig. 4 shows the induction profiles of the two promoters in response to increasing concentrations of the inducers. In the case of the *Ptrc*-based construct, the promoter activity grew in response to the increased dosage of IPTG and reached maximal levels at concentrations equal to or above 250 µM (Fig. 4a). Additionally, this system reached a maximal expression level after 6–8 h of induction. When the *Pb* fusion was analyzed in *P. putida*, we observed that maximal activity was reached upon exposition to a higher inducer concentration than in the first case, as 500 µM of benzoate was necessary to fully induce the system (Fig. 4b). This induction profile was nearly identical to two previous analyses using a *Pb* fusion to the *lux* operon [30], [32]. Furthermore, the time required to reach the maximal expression levels was in the same range as for the *Ptrc* fusion. These results validate the applicability of the dual GFP-*luxCDABE* cassette for the analysis of promoter induction kinetics over a wide range of inducer concentrations.

### Single-cell Analysis Using the GFP Reporter

**Figure 5:**
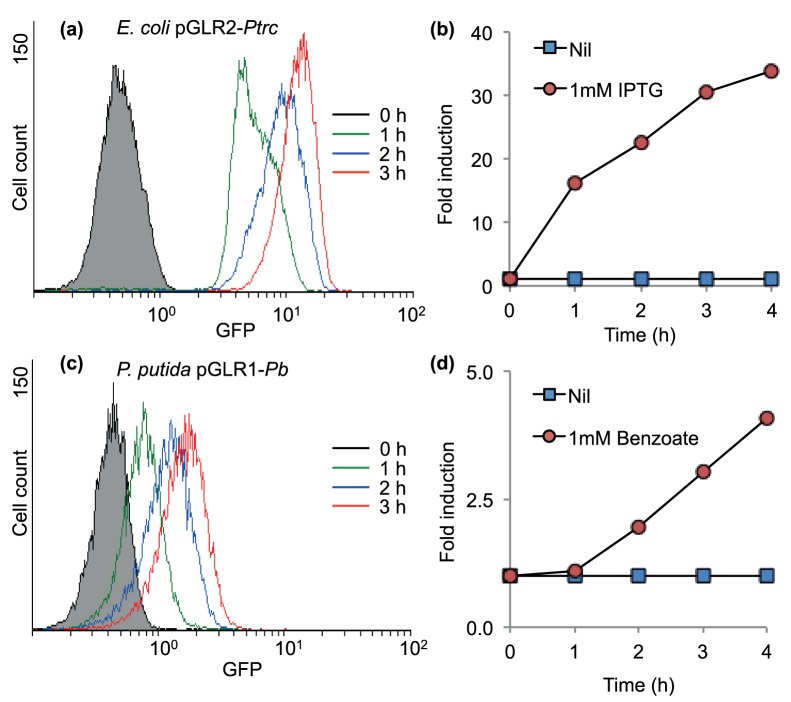
Single-cell analysis of *Ptrc*- and *Pb*-based systems. Overnight-grown strains were diluted 1∶20 in fresh media and allowed to grown to mid-exponential phase. At this point, 1 mM of IPTG for *E. coli* harboring the *Ptrc::*GFP*-luxCDABE* system (**a**) or 1 mM of benzoate for *P. putida* with *Pb::*GFP*-luxCDABE* (**b**) were added to the culture. At time intervals of 1 hour, samples were collected and stored on ice until analysis by flow cytometry. Untreated cells were used as controls. For each assay, 15,000 cells were analyzed. (**c**) Induction profile of *E. coli Ptrc::*GFP*-luxCDABE* strain in response to 1 mM of IPTG. (**d**) Induction profile of *P. putida Pb::*GFP*-luxCDABE* strain in response to 1 mM of benzoate. Profiles in (**c**) and (**d**) were calculated by normalizing the average fluorescence levels of induced populations by fluorescence levels of the control samples with no treatments.

As shown in the previous section, the *lux* operon facilitates the quantification of promoter induction kinetics under a wide range of assay conditions (such as in the presence of different inducer concentrations). However, another relevant piece of information about gene regulation is the performance of the system at the single-cell level. In general, promoters in single cells behave in either a graded fashion or an all-or-none fashion [33], [34]. In graded behavior, all cells in the population switch to the ON state (where the promoter is active) upon sensing the cognate signal [35]. In this system, the observed change in the expression level at the population scale faithfully reflects the accumulation of the transcript/reporter in individual cells [36]. Conversely, cells can display bimodal behavior where some individuals turn to the full ON state while others remain in the OFF state. In this case, the changes in the population expression levels reflect variations in the relative proportion of cells, which are ON *vs.* OFF [37]. Thus, in this last scenario, a mixture of cells with active and inactive promoters coexists in a culture with inducer. Several reports have provided examples of both modes of operation along with the key determinant factors necessary to generate this behavior in some particular cases [5], [33], [35], [36]. To validate the potential of the dual GFP-*luxCDABE* reporter cassette as a tool for diagnosing the single-cell behavior of target promoters, we examined the expression profiles of the *Ptrc*- and *Pb*-based system in response to 1 mM of IPTG or benzoate, respectively, through the analysis of the GFP reporter. To this end, overnight-grown cells (i.e., *E. coli* with pGLR2-*Ptrc* and *P. putida* with pGLR1-*Pb*) were diluted in fresh media and allowed to grow until mid-exponential phase. At this point, each reporter strain was exposed to the specific inducer and incubated for several hours. At time intervals of 1 h, samples were taken and analyzed by flow cytometry as indicated in Material and Methods. As shown in Fig. 5a and Fig. 5c, both expression systems exhibited graded behavior at the single-cell level. These results are in agreement with previous reports for the behavior of *Ptrc* [38] and *Pb* [30] systems, revealing that the synthetic vectors used to implement the dual reporter cassette do not interfere with the native activities of the target promoters. Moreover, the comparison of the induction dynamics of the two expression systems (Figs. 5b and 5d) demonstrated a profile similar to that observed using the *lux* operon as described in the previous section whereby reporter is strongly activated during the first hours of induction. Finally, the results presented in Fig. 5 highlight the utility of the dual GFP-*luxCDABE* reporter system for studying single-cell behavior in gram-negative bacteria.

### Noise Quantification for Mono-copy and Multi-copy Reporter Systems

**Figure 6:**
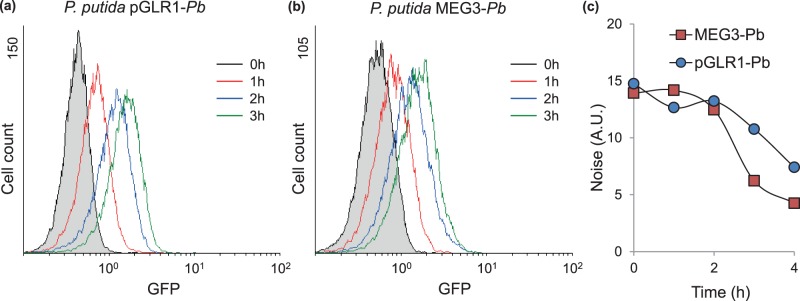
Comparison of mono-copy *vs.* multi-copy reporter systems. Briefly, overnight-grown strains were diluted 1∶20 in fresh media and allowed to grown to mid-exponential phase. At this point, 1 mM of benzoate was added to *P. putida* MEG3-*Pb* (**a**) or *P. putida* with *Pb::*GFP*-luxCDABE* (**b**). At time intervals of 1 hour, samples were collected and stored on ice until analysis by flow cytometry. Untreated cells were used as controls. For each assay, 15,000 cells were analyzed. (**c**) Noise quantification in mono-copy and multi-copy reporter system. Squares represent the data from experiments shown in (**a**)**,** while circles are for experiments in (**b**).

The dual reporter cassette implemented here allowed us to inspect both the population and the single-cell behavior of the two assayed systems. However, one aspect of the experimental setup with the GFP-*luxCDABE* cassette is its implementation with broad-host range plasmid vectors based on pBBR1 and RK2, two of the most promiscuous replication origins [23]. As autonomous entities capable of replicating independently of the host chromosome, plasmids facilitate cloning operations [34] but they can also increase noise during the expression of the assayed promoter [39], [40]. The pBBR1 and RK2 based vectors are expected to be present at 30–40 and ∼11 copies per cell, respectively [41], [42]. However, these vectors are much less abundant in the cell than are typical cloning plasmids based on the ColE1 origin, which can be found at about 300–1000 copies per cell [43]. Thus, the reduced number of copies of the pGLR1/2 vectors are expected to increase little –if anything the noise level of the promoter under examination. To assess the copy number effect on the performance of the GFP*-lux* reporter, we compared the levels of noise generated during *Pb* activation in *P. putida* harboring the pGLR1-*Pb* vector *vs.* another reporter strain in which an equivalent *Pb*::GFP fusion is placed in monocopy in the chromosome of *P. putida* MEG3-*Pb* [30]. This strain has a *Pb* promoter sequence fused to a bi-cistronic GFP-*lacZ* fragment that was used to investigate stochastic processes during benzoate degradation in *P. putida* [30]. For the comparison we chose to use pGLR1-*Pb,* as it is based on the pBBR1 origin that has a higher copy number per cell [41]. Furthermore, in this test system, the *benR* gene (encoding the activator for *Pb*) is placed in monocopy in the chromosome; thus, any interference occasioned by the presence of multiple target promoters should be enhanced. The induction experiments were performed in response to 1 mM of benzoate, and cells were analyzed by flow cytometry as before. As shown in Fig. 6a, *P. putida* harboring pGLR1-*Pb* presented a single-cell profile very similar to that from *P. putida* MEG3-*Pb* (Fig. 6b) assayed under identical conditions. To quantitatively compare the level of stochastic variation from cell to cell in the two reporter strains, we calculated the noise for each time point for the experiments in Fig. 6a and 6b. Noise was determined as described previously [44] and it is obtained by dividing the variance in the fluorescence level across the population by the average fluorescence in the whole sample (see the Material and Methods for more details on the noise calculation). As shown in Fig. 6c, despite minor differences, the mono-copy and multi-copy reporter systems presented comparable levels of noise during *Pb* activation in response to benzoate. These findings demonstrate that the plasmid reporter systems implemented here contribute very little to the stochasticity of the assayed promoters, and thus, we advocate this tool as a valuable asset for investigating gene expression at the single-cell level in bacteria.

### Conclusion

The work presented here shows the implementation and validation of a novel GFP-*luxCDABE* dual reporter system that is suitable for promoter probing in gram-negative bacteria beyond *E. coli.* Although other GFP*-lux* constructs have been reported in the literature [20], they seem to be optimized for specific gram-positive hosts and they have not worked well in our hands. As this new system is based on broad-host range vectors, it can be used in principle in a wide variety of strains. In addition to *E. coli* and *P. putida*, these vectors are expected to replicate in bacteria from the genera *Alcaligenes*, *Bordetella*, *Caulobacter*, *Rhizobium*, *Rhodobacter*, *Vibrio* and *Xanthomonas*, among many others [23]. Additionally, the advantages of the combination of the two reporters, GFP and *lux*, into one single cassette makes this dual system a powerful tool for investigating gene regulatory networks under native conditions using a simplified cloning step. Moreover, the presence of the reporters in the multi-copy vectors presented here does not significantly increase the noise generated during promoter regulation in respect to a chromosomally located system [30]. Finally, we expect that this new tool will have a significant impact not only in the field of Molecular Biology but also for Systems and Synthetic Biology applications in standard and alternative bacterial hosts.

## Materials and Methods

### Strains, Plasmids and Growth Conditions

The strains and plasmids used in this work are listed in Table 1. *E. coli* CC118 was used as the host strain for all the cloning procedures, and *E. coli* MG1655 was used as the host for the *PlexA* GFP-*luxCDABE* construct. Broad-host range plasmids were transferred to *P. putida* KT2440 by tripartite mating as previously described [45]. *E. coli* strains were grown at 37°C in Luria-Bertani (LB) medium or in M9 minimal medium supplemented with 0.4% (w/v) glucose, 0.1% (w/v) casamino acids, 2 mM MgSO_4_, 0.1 mM CaCl_2_ and 0.05% (w/v) vitamin B1. *P. putida* cells were incubated at 30°C in M9 minimal medium supplemented with 2 mM MgSO_4_ and 25 mM succinate as the sole carbon source. When required, kanamycin (Km, 50 µg mL^−1^) or chloramphenicol (Cm, 30 µg mL^−1^) was added to the growth media.

### General Cloning Procedures

Cloning procedures were performed as described previously [46]. DNA was amplified with the polymerase chain reaction (PCR) by mixing 50–100 ng of the template with 50 pmol of each primer and 2 U of GoTaq polymerase (Promega) in a 100 µL reaction volume. The mixture was then subjected to 25 cycles of 4 min at 95°C, 30 s at 53–60°C and 1 min at 72°C. Primers and benzoate were purchased from Sigma-Aldrich. To assemble the dual reporter system, the GFP variant from the pGreenTIR vector [24] was PCR amplified using primers 5-GFP (5′-GCG GCT GCA GGC ATG CAG GAG GAA AAA CAT ATG AGT AAA GG-3′) and 3-GFP (5′-GCG GAA GCT TCT ATT TGT ATA GTT CAT CCA TGC C-3′), generating a fragment of 767 bp. This fragment was then cloned as a *Pst*I/*Hind*III fragment into pSEVA236 (a promoter probe vector with the pBBR1 origin of replication), previously digested with the same enzymes. The resulting vector was named pGLR1 and has a tandem GFP*-luxCDABE* fragment downstream of a multiple cloning site (Fig. 1a). The DNA sequence of the short-lived *gfp lva* variant [26] was similarly amplified by PCR using primers 5-GFP-lva (5′- GCG GCT GCA GGC ATG CAG GAG GAA AAA CAT ATG CGT AAA GG-3′) and 3-GFP-lva (5′- GCG CAA GCT TTT AAG CTA CTA AAG CGT AGT TTT CG -3′), generating a fragment of 788 bp. This fragment was then cloned as a *Pst*I/*Hind*III fragment into pSEVA236, digested with the same enzymes as before. The resulting vector was named pGFLR1 and has, as previously described for pGLR1, the GFPlva*-luxCDABE* fragment located downstream of a multiple cloning site. The construction of pGLR1-*PlexA* involved the amplification of the 106 bp fragment *PlexA* promoter [27] with primers 5PLEX (5′-CCC TTC CAG AAT TCG ATA AAT CTC TGG-3′) and 3PLEX (5′-CCC GGA TCC TCC GCC CCC TGG GTG TAT ATA CAG-3′), with the amplified sequence cloned as an *EcoRI*/*BamHI* fragment into pGLR1. The same *PlexA*-containing *EcoRI/BamHI* fragment was cloned in pGFLR1, thus creating pGFLR1-*PlexA*. For the cassette encoding the stable GFP variant, the ∼6.6 Kb fragment of pGLR1 spanning the GFP*-luxCDABE* segment was excised with *Sph*I/*Spe*I restriction enzymes and cloned into a pSEVA221 (a broad-host range vector based on the RK2 origin [25]), generating vector pGLR2. To validate the dual reporter system, two different promoters were assayed. First, the *Ptrc*-based expression system of pSEVA224 [25] was cloned into pGLR2 as a *Pac*I/*Avr*II fragment, generating the pGLR2-*Ptrc* plasmid. Similarly, a ∼500 bp *Pb* promoter from the benzoate degradation pathway of *P. putida* [29] was PCR amplified using primers 5-PB (5′-TGG ATG AAT TCG ACA GTA CCC TCC-3′) and 3-PB (5′-GCG CGG ATC CGG CCA GGG TCT CCC TTG-3′) and cloned as an *Eco*RI/*Bam*HI fragment into pGLR2, generating the pGLR2-*Pb* plasmid. This construct was then mobilized into *P. putida* strain KT2440, which has all native regulatory elements necessary to trigger *Pb* promoter activity in response to benzoate [29]. The correctness of the cloned fragments was confirmed by DNA sequencing in all cases.

To check for potential interference between the two reporter systems when they were placed in tandem, two additional plasmids where constructed in which each reporter was placed alone under the control of an inducible promoter (i.e. *Ptrc*). In one case, the *gfp tir* gene was cloned as a *Pst*I/*Hind*III fragment into pSEVA224 [25], generating the pGFP-*Ptrc* plasmid, while in the other case, the *luxCDABE* operon was cloned as a *Hind*III/*Spe*I fragment into the same pSEVA224, resulting in the pLUX-*Ptrc* plasmid. The resulting plasmids were introduced in *E. coli* CC118, and the resulting reporter strains were analyzed as described below.

### Bioluminescence and Fluorescence Assays of Whole Populations

Emission of fluorescence and production of light in cells growing on Petri dishes were recorded with a luminometer VersaDoc imaging system Model 4000 (Bio Rad), and the images were captured and processed with the analysis software Quantity One 4.6.9 of the same brand. For quantitative promoter activity assays, single colonies of *E. coli* and *P. putida* reporter strains were picked from fresh plates and inoculated into 5 mL of minimal medium with corresponding antibiotics. The cells were then grown overnight at 170 rpm. After pre-growth, the cells were washed twice with 10 mM MgSO_4_ buffer and diluted 1∶20 (v/v) into fresh medium with different concentrations of IPTG (in the case of the *Ptrc*-based system) or benzoate (for the *Pb*-based reporter). Microtest™ 96-well assay plates (BD Falcon) were filled with 200 µL per well of diluted culture and placed into a Wallac Victor 2 Microplate Reader (Perkin Elmer). Plates were then incubated with shaking for several hours. At time intervals of 30 min, the optical density at 600 nm (OD_600_) and the luminescence or the fluorescence of each of the cultures were measured. Non-inoculated M9 medium was used as a blank for adjusting the baseline for measurements. Promoter activities were calculated by normalizing the reporter signals (luminescence or fluorescence) to the OD_600_ readings. Data processing was performed using MATLAB software (MathWorks).

### Single-cell Analysis by Flow Cytometry

Single-cell experiments were performed with a Gallios (Perkin Elmer) flow cytometer. To this end, GFP was excited at 488 nm, and the fluorescence signal was recovered with a 525(40) BP filter. Overnight-grown cells were diluted 1/20 in fresh M9 media containing the carbon source indicated in each case and incubated for 4–5 hours. After this pre-incubation, at the mid-exponential phase, the cells were split into two samples: one was induced by the corresponding compound and the other was used as a non-induced control. Cultures were then incubated with shaking in air at the appropriate temperature, and each hour after induction, an aliquot of each sample was stored on ice until analysis. For every aliquot, 15,000 events were analyzed. The data processing was performed using Cyflogic software (http://www.cyflogic.com/).

### Noise Quantification

The noise for the single-cell experiments was calculated as previously described [44]. Noise is generally defined as the level of cell-to-cell variation in the fluorescence signal normalized by the average fluorescence in the population. For the noise calculation, the flow cytometer output files generated during single-cell analyses were converted from listmode (.lmd) to ASCII text files using LLDATA software (http://www.cyto.purdue.edu/flowcyt/software/Catalog.htm). Next, individual fluorescence values were processed with the R package (http://www.r-project.org/) to calculate the variance (*var*) and mean values (*mean*) of the samples. Finally, the noise values for each experiment were determined simply by dividing the *var* by the *mean* values.
